# Home-run trials for rare cancers: giving the right drug(s) to the right patients at the right time and in the right place

**DOI:** 10.1038/s41698-023-00487-5

**Published:** 2023-12-08

**Authors:** Jacob J. Adashek, Razelle Kurzrock

**Affiliations:** 1 Department of Oncology, The Sidney Kimmel Comprehensive Cancer Center at The Johns Hopkins Hospital, Baltimore, MD USA; 2 WIN Consortium, Paris, France; 3 MCW Cancer Center, Milwaukee, WI USA; 4 University of Nebraska, Omaha, NE USA

**Keywords:** Cancer, Cancer genomics

## Abstract

In oncology clinical trials, many patients spend their final months at a central clinical trial facility far from home for “mandatory” protocol visits/diagnostic testing. Studies suggest that the travel strain may be greatest among patients living in low‐income areas and/or participating in early-phase studies. In this regard, rare cancers constitute a special unmet need with limited therapeutic options and few trials. Though individually uncommon, rare cancers as a group constitute ~22% of the cancer burden; the portion of cancer burden may even be greater if biomarker-defined rare subsets of either a single cancer type or a tissue-agnostic subgroup are included. Exacerbating the access issue is the fact that, in addition to the paucity of trials, many centers will not activate existing single-arm trials, often due to accrual concerns, which may further disadvantage this patient group and also jeopardize trial completion. Decentralized clinical trials may resolve some of these challenges by allowing patients to participate from close to home. Decentralized clinical trials can take the form of being site-less, with the coordinating body working remotely and care provided by the home oncologist, or by taking the tack of National Cancer Institute/cooperative groups (e.g., NCI-MATCH genomics matching trial or SWOG1609 [NCI] DART immunotherapy rare cancer trial) using a platform design with multiple cohorts and opening at >1000 sites. Decentralized trials now also have supportive FDA guidance. Importantly, home-run trials permit clinical trial access to underserved groups, including those in rural areas and patients financially unable to travel to a central facility.

## Introduction

**Fig. 1:**
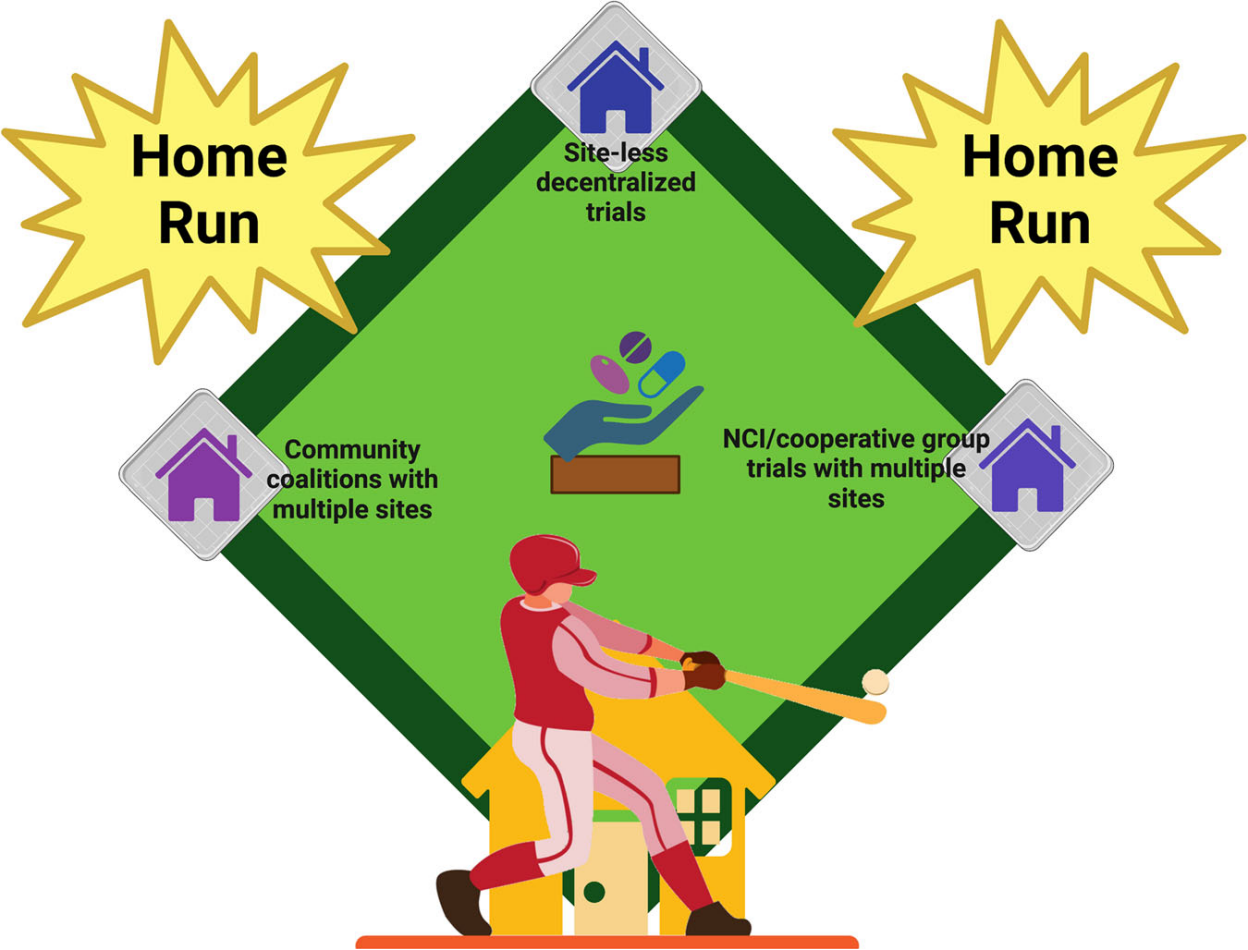
Trials from home are home run trials. Baseball diamond with therapies (pitcher’s mound) getting drugs to patients via site-less decentralized remote trials, community coalitions and NCI/cooperative groups.

Amongst the goals of precision medicine clinical trials is to give the right drugs to the right patients at the right time^1^. Yet, a major challenge for patients with rare cancers is accessing clinical trials, i.e., trial availability in the right place – close to home (Fig. 1). Indeed, most clinical trials are open at a limited number of centers, often far from the patient’s residence^2^. As a result, patients incur hardship at multiple levels, including the financial cost of travel and the human cost of being away from home and family when they are ill.

There are more than 500,000 interventional clinical studies registered globally, involving tens of thousands of investigators, and hundreds of thousands of patients (https://www.statista.com/statistics/732997/number-of-registered-clinical-studies-worldwide/#:~:text=Clinical%20studies%20are%20an%20important, t. c. s. r. g.), (https://www.who.int/observatories/global-observatory-on-health-research-and-development/monitoring/number-of-trial-registrations-by-year-location-disease-and-phase-of-development#data-sources), (https://clinicaltrials.gov/ct2/resources/trends)^3^. In oncology alone, there are ~2300 trial starts yearly and ~83,000 registered interventional trials (https://www.statista.com/statistics/1092600/oncology-clinical-trial-starts-worldwide/). In cancer clinical trials, many patients spend precious last months of their lives at a central clinical trial site in a distant city for scheduled study visits and diagnostic testing^4^. The burden of travel may be highest among patients enrolled in early-phase studies or living in low‐income areas^5^. Moreover, there has been a rapid rise in trial monitoring requirements and emphasis on patient compliance with complex protocols^6^.

The COVID pandemic accelerated adoption of remote videoconferencing solutions in business and for cancer patients^7,8^. Even so, telehealth-based options are not yet fully enabled for clinical trial execution. Virtualizing clinical trial study visits are important patient-centric tactics that would attenuate participant burden.

The FDA defines a decentralized clinical trial (DCT) as “some or all of a clinical trial’s activities occur at locations other than a traditional clinical trial site. These alternate locations can include the participant’s home, a local health care facility, or a nearby laboratory” (https://www.fda.gov/drugs/news-events-human-drugs/evolving-role-decentralized-clinical-trials-and-digital-health-technologies#:~:text=In%20a%20decentralized%20clinical%20trial, f. C.) and the Clinical Trials Transformation Initiative “defines DCTs as those in which some or all study assessments or visits are conducted at locations other than the investigator site via any or all of the following DCT elements: tele-visits; mobile or local healthcare professionals, including local labs and imaging centers; and home delivery of investigational products. Decentralized clinical trials can be completely remote or partially decentralized with hybrid approaches. Hybrid trials are those that require some visits to be conducted on site, while other visits or assessments can be performed at a participant’s home or within their local care community. Fully remote trials have no required site visits” (https://ctti-clinicaltrials.org/wp-content/uploads/2022/04/CTTI-Digital-Health-Trials-Planning-Decentralized-Trials-Recs.pdf).

A home-run therapeutic clinical trial allows patients to be treated with novel therapeutics at home, either through decentralized, site-less patient care, and/or through having very large numbers of sites throughout the country (Table 1 [https://investor.hcahealthcare.com/news/news-details/2022/McKesson-and-HCA-Healthcare-Announce-Plans-to-Form-an-Oncology-Research-Joint-Venture-to-Advance-Cancer-Care-and-Increase-Access-to-Oncology-Clinical-Research/default.aspx]). Herein, we discuss models for home-run trials, especially those that address patients with rare and ultra-rare cancers.

**Table 1:** Examples of home-run types of initiatives that focus on or include rare cancers.

Type of Sponsor	Trial	Organizing Body/Sponsor	Comment	Reference/NCT number
NCI Division of Cancer Treatment and Diagnosis	NCI MATCH	NCI at the NIH	Platform trial including multiple genomically matched tissue-agnostic cancers >1000 sites at its peak	NCT02465060
NCI SWOG Early therapeutics and rare cancers committee	DART Trial (SWOG1609)	SWOG/NCI	Platform immunotherapy (nivolumab and ipilimumab) trial for multiple rare cancers defined by their histology >1000 sites at its peak	NCT02834013
TargetCancer Foundation	TRACK trial	TargetCancer Foundation (non-profit foundation	Precision medicine (N-of-1) trial based on next generation sequencing and individually matching of patients with rare cancers to treatment (Decentralized, completely site-lss remote Patient cared for by home oncologist	NCT04504604
Alpha T trial	Alpha-T Trial	Science37/Roche (Industry)	Decentralized clinical trial involved a tumor-agnostic approach (alectinib for *ALK*-altered cancers) Decentralized, completely site-less remote) Patient cared for by home oncologist	NCT04644315
Community Practices	Multiple trials	Sarah Cannon/US Oncology Network (McKesson Corporation and HCA Healthcare) (For-profit corporations)	~60 research sites and >170 locations and managing ~500 active trials at any given time 182 hospitals and ~2300 ambulatory sites of care	https://investor.hcahealthcare.com/news/news-details/2022/McKesson-and-HCA-Healthcare-Announce-Plans-to-Form-an-Oncology-Research-Joint-Venture-to-Advance-Cancer-Care-and-Increase-Access-to-Oncology-Clinical-Research/default.aspxhttps://investor.hcahealthcare.com/news/news-details/2022/McKesson-and-HCA-Healthcare-Announce-Plans-to-Form-an-Oncology-Research-Joint-Venture-to-Advance-Cancer-Care-and-Increase-Access-to-Oncology-Clinical-Research/default.aspx

### The unmet clinical trials’ need in rare cancers

Rare cancers are an underserved oncology area. They are variably defined, but often are designated “rare” when the incidence is fewer than six per 100,000 people (https://www.rarecancerseurope.org/what-are-rare-cancers/definition-of-rare-cancers). By definition, rare cancers are individually uncommon, yet, in total, they constitute ~22% of the cancer burden, and there are ~200 rare cancer types^9^. Despite this, rare cancers see a minuscule amount of funding when compared to common cancers^10^. Furthermore, rare/ultra-rare cancers occur more frequently among younger and nonwhite persons, and their prognosis tends to be worse, possibly because of the relative paucity of expertise, approved therapies, and clinical trials^9,11^. Even so, rare cancers can sometimes be successfully addressed with properly targeted therapies^12^. Hence, patients with rare cancers may be interested in enrolling in clinical trials. However, there may be a paucity of clinical trials for their rare/ultra-rare cancer, and the few clinical trials that exist may be available at only a limited number of sites, especially because many centers are reluctant to open studies that will not accrue well; the latter harms both the chance for trial completion and imposes additional difficulties for patients with rare cancers.

### Tissue-agnostic biomarker-driven trials – a different type of “rare” cancer

Rare cancers are generally defined by their incidence (<6/100,000) and by their histology. However, there is a different and at least equally important type of rare cancer, defined by rare tissue-agnostic genomic events^13^. These rare tumors can be of any histology or originate in any organ, but they are connected by a common molecular abnormality. When properly targeted, their response rates are often high and, to date, there are several tumor-agnostic biomarker-based FDA approvals in oncology, established because of response rates of ~30–75%^13^.

## National Cancer Institute (NCI) initiatives

Because there is universal agreement that rare cancers represent an unmet medical need, the NCI Center for Cancer Research moonshot goals include building a rare tumor patient engagement network, creating a comprehensive oncology network to evaluate rare central nervous system tumors (NCI-CONNECT) as well as forming a moonshot pediatric, adolescent, and adult rare tumors network (MyPART) https://ccr.cancer.gov/research/cancer-moonshot. Further, the NCI-MATCH trial (discussed below). represents a crucial countrywide therapeutic initiative for patients with a variety of cancers. Moreover, the NCI Director (and now nominated head of the National Institutes of Health [NIH]) Monica Bertagnolli has made “rare cancers” a mandate within her national cancer plan goals (https://nationalcancerplan.cancer.gov/goals). The underlying understanding is that access may be a particular problem for patients with rare cancers, and the issue is especially important for underrepresented minority patients and for patients in rural communities for whom clinical trial availability is a major concern in the context of both common and rare diseases.

## Platform trials (a clinical trial design innovation)

### NCI MATCH (NCT02465060)

#### A national platform trial for genomically matched tumor groups

The National Cancer Institute–Molecular Analysis for Therapy Choice (NCI-MATCH) trial was the first national-scale platform clinical trial in the USA including centralized diagnostic testing and geographically distributed clinical investigation of multiple treatment options in parallel^14^. NCI-MATCH opened in August 2015; 1117 sites enrolled 1,201 patients on 38 cohorts; the trial closed in December 2022. The NCI Central Institutional Review Board for Early Clinical Trials was the institutional review board of record. The study enabled many tumor-agnostic accruals targeting uncommon genomic events with gene-specific targeted therapies. There were several matches, such as targeting *PIK3CA* with copanlisib (but not with taselisib) and targeting *AKT1* E17K-mutated tumors with capiasertib that met the study response endpoints^15–17^. However, targeting *NF1*, *GNAQ*, or *GNA11* with trametinib alone, CCND1, 2, or 3 amplifications with palbociclib-alone, *NRAS*-mutated cancers with binimetinib, or *ERBB2* single-nucleotide variants or insertions/deletions with afatinib did not meet their primary endpoints^18–21^. NCI-MATCH demonstrated the feasibility of nationwide accrual of patients on a platform trial with multiple rare genomic basket subgroups across numerous histologies, with successful biopsy and enrollment at local facilities^14^.

### Southwest Oncology Cooperative Group (SWOG) and the DART trial (SWOG1609 [NCI] NCT02834013)

#### A national immunotherapy platform trial for rare cancers

The SWOG Cancer Research Network’s DART (Dual Anti-CTLA-4 and Anti-PD-1 Blockade in Rare Tumors) trial is run through SWOG’s Early Therapeutics and Rare Cancer Committee; it activated in January 2017 and included 53 cohorts of rare cancers. This study aimed to fill the void wherein patients with rare cancers are ineligible for immune checkpoint inhibitor trials. The DART trial used the same single-arm two-stage phase II design for each cohort, with the primary endpoint being RECIST response. Importantly, there was also a “Not Otherwise Categorized” cohort. Although many institutions may not have opened trials for a single rare histology, by activating DART, institutions were essentially opening 53 trials in one protocol. At its peak, the DART study was open at >1000 sites across the NCI’s SWOG National Cooperative Trial Network, making the study accessible to patients across the USA. All SWOG/NCI sites are pre-approved before membership in the SWOG cooperative group is confirmed and they are audited regularly to ensure their ability to comply with protocol requirements.

To date, four cohorts of rare/ultra-rare cancers (metaplastic breast cancer, angiosarcoma, and two cohorts of neuroendocrine tumors) have been published in the peer-review literature and show a subset of highly immunotherapy-responsive patients^22–25^. Approximately 10 additional cohorts of rare/ultra-rare cancers have been presented at major national meetings and/or in abstract form, and some of the published cohorts are now impacting NCCN guidelines.

## Decentralized clinical trials (an operational approach to delivering clinical trials)

### TargetCancer Foundation and the TRACK trial (NCT04504604)

#### A national precision genomics (N-of-1) remote, site-less trial for rare cancers

The TargetCancer Foundation TRACK (Target Rare Cancer Knowledge) study is a patient advocacy-initiated, non-randomized, fully remote study that is a unique example of a national home-run clinical trial for precision genomics in rare cancers. It aims to establish if patients with rare tumors benefit from individually matched molecular therapy as dictated by their clinical-grade next-generation sequencing (NGS) (https://www.foundationmedicine.com/test/foundationone-cdx). The accrual goal is 400 patients. The protocol is approved by a central Internal Review Board and consent is obtained remotely. The TargetCancer Foundation trial staff ensure medical record collection, contact with patient and their physician, and mobile phlebotomy to collect liquid biopsies, as well as obtaining existing tissue samples for NGS testing. NGS informs the treatment recommendations provided by a panel that includes expert oncologists (medical and surgical), clinical trialists, pathologists, molecular genomics specialists, pharmacist, and a genetic counselor as part of a weekly cross-country (USA) virtual Molecular Tumor Board (MTB) coordinated by the TargetCancer Foundation. A medication acquisition specialist can assist with procuring suggested agents. MTB suggestions may include navigation to secondary clinical trials or FDA-approved drugs. Insight from previous combination approach trials such as I-PREDICT often inform the treatment algorithms^26–30^. The home physician chooses and delivers treatment. The study opened in October 2020 and by June 2023 enrolled 141 patients from 37 states.

## Industry Initiatives and the Alpha-T trial (NCT04644315)

A unique decentralized clinical trial involved a tumor-agnostic approach using the FDA-approved oral ALK inhibitor alectinib to target *ALK* fusions (since prior anecdotes suggested responsiveness), with *ALK* fusions being an ultra-rare genomic alteration found in only ~0.2% of malignancies outside of non-small cell lung cancers (NSCLCs)^31,32^. This trial was home-based, with the patient cared for by their treating oncologist (partnering with trial investigators to ensure protocol compliance). Patients received at-home care assessments via telehealth and clinical trial nursing staff. Patients were identified as qualifying via the Foundation Medicine Inc. NGS platform (https://www.foundationmedicine.com/) and, if the treating clinician approved, they were connected to a remote trial team including a Science37 investigator (since Science37 ran the trial) (https://www.science37.com/) and offered enrollment/consented remotely once the central study team confirmed eligibility^33,34^. Clinical research coordinators/nurses visited patients at home to collect end-point data through physical examination, phlebotomy, and questionnaires. A trial investigator completed clinical assessments using telemedicine tools. The study used local radiology facilities. The remote trial team was responsible for training, onboarding and data collection as well as selection of/contracting with phlebotomy/imaging/home health services^34^. The trial allowed patients, even in rural areas far removed from tertiary cancer centers, to receive a cutting-edge therapy .

In some ways, the trial was especially well-suited to a decentralized, home-run model: (i) the targeted alteration was ultra-rare but could be identified by an NGS assay used nationally; and (ii) the ALK inhibitor tested – alectinib – was already FDA-approved (for the treatment of ALK aberrant NSCLC)^32^, is oral, and is well tolerated. On the other hand, the extreme rarity of the alteration made trial accrual challenging^35^.

## Changes in the management and delivery of oncology care in the community

### Role of community practices

Approximately 85% of cancer patients are treated in the community and not in large academic centers^36^. The merging of groups such Sarah Cannon Research Institute, a world-renowned cancer clinical trials organization, and US Oncology, a very large community oncology practice, seeks to close this gap. Other examples are clinical trial groups such as NEXT Oncology, venturing with Virginia Cancer Specialists and Texas Oncology (https://nextoncology.com/next-oncology-expands-phase-i-program-with-vcs-partnership/). These sizeable coalitions of community practices with large-scale clinical trial portfolios may streamline access for patients who are seen by their community oncologist, without having the physical and financial burdens of leaving one’s familiar specialist and traveling to academic centers located far from the patient’s home.

### Feasibility of home-run care

Home-based cancer care is safe, feasible, and saves costs^37^. Between 2015 and 2019, the Christie NHS Foundation Trust, Europe’s largest single-site cancer center, provided >11,900 in-home injections or intravenous infusions via a trained nurse, with laptops enabling real-time documentation^37^. The United States Department of Veterans Affairs National Oncology Program manages the largest integrated hematology and oncology services in America, providing care for >43,000 veterans yearly; they have expanded access to cancer treatments through the “Close to Me” program^38^. From October 2021 to December 2022, 373 veterans were safely treated at Close to Me clinics, which saved ~43,200 patient drive miles and >$813,350 in medication costs, and boasted almost universal treatment adherence^38^.

## Guidance from the Food and Drug Administration (FDA)

The FDA has fully embraced the concept of decentralized trials and, in spring 2023, published their own guidance (https://www.fda.gov/regulatory-information/search-fda-guidance-documents/decentralized-clinical-trials-drugs-biological-products-and-devices). The guidance permits patients to participate in trial-related activities in part or fully from home or a local healthcare facility, but recommends that all trial-related records be housed centrally. The FDA also understands the need for video visits. In the instances where an in-person visit would be necessary, trial personnel could be sent to the patient’s home or local facility, or local physicians or nurses could be asked to perform a physical examination, obtain vital signs, or read radiographic reports on a fee-for-service basis paid for by the sponsor. Standardization for decentralized trials in regard to outcome measures and adverse event recording can be defined in the protocol and be performed and recorded locally (or via study investigators who access the information remotely) and the information transmitted to a central site.

## Future directions: emerging options for home-run decentralized trials

Clinical trial access is especially important for patients with rare cancers, who represent an unmet need in that their cancers often have few approved therapies and even fewer clinical trial options. Decentralized therapeutic platform trials for rare cancers have been embraced by a cooperative group (SWOG1609 [NCI] and the DART immunotherapy trial) as well as the NCI-MATCH genomics matching trial (both having been open at >1000 sites), a non-profit foundation (TargetCancer and the TRACK precision genomics trial), and a for-profit endeavor (Science37 Alpha-T *ALK* fusion targeting tissue-agnostic trial) (with the latter two using fully site-less care provided by a home oncologist). Improving access for patients with cancer to clinical trials and to novel therapeutics is also endorsed by the NCI Director in her national plan. Moreover, extremely large networks of community oncologists such as those resulting from US Oncology Research and Sarah Cannon Research Institute merger offer the potential for local clinical trials. Decentralized trials now also have supportive guidance from the FDA. Importantly, home-run trials permit clinical trial access to underserved groups in rural areas and for those financially unable to travel to a central facility. As a future work, one can build a standard operating procedure with the regulatory bodies to solidify the concept of home-run trials where these can be the go-to option for every rare cancer, so we do not deplete resources just launching these rare cancer studies only at a few centers.
